# Socioeconomic differentials in the association between hysterectomy and hypertension among women in Maharashtra

**DOI:** 10.3389/frph.2026.1783545

**Published:** 2026-04-17

**Authors:** Jitendra Gupta, Aamishi Mishra

**Affiliations:** 1 Department of Community Medicine, Symbiosis Medical College for Women, Symbiosis International University, Lavale, Pune, India; 2 Symbiosis Medical College for Women, Symbiosis International University, Lavale, Pune, India

**Keywords:** gynecological, hypertension, hysterectomy, obesity, poor

## Abstract

**Background:**

Hysterectomy is a commonly performed gynecological surgery for conditions such as uterine fibroids, endometriosis, and abnormal uterine bleeding. However, the procedure provides therapeutic benefits, its increasing prevalence at older reproductive ages of women has raised concerns regarding long-term health outcomes, particularly hypertension and the associated risk of cardiovascular disease (CVD). This study explores the relationship between hysterectomy and hypertension, and acts as a contraindication among reproductive-aged women in Maharashtra.

**Methods:**

This study analysed data from the National Family Health Survey (NFHS-5, 2019–21) for 33,755 women aged 15–49 years in Maharashtra. Descriptive, Oaxaca decomposition and comparative analyses were conducted to assess the prevalence of hysterectomy among hypertensive and overall general women across socio-demographic, reproductive, and health-related characteristics.

**Results:**

The prevalence of hysterectomy among women with hypertension was 7.8 percent, more than double the overall prevalence among all women (3%). Higher prevalence was observed among women aged 40–49 years and those residing in rural areas. The majority of hysterectomies were performed in private healthcare facilities. Increased prevalence was associated with lower educational attainment, lower wealth quintiles, higher parity, obesity (11.6%), and female sterilization (13.7%) compared to their respective counter parts. Abnormal uterine bleeding emerged as the most common indication for hysterectomy, followed by uterine fibroids. The findings indicate a complex and potentially association between hysterectomy and hypertension.

**Conclusion:**

The research study highlights a significant association between hypertension and hysterectomy among reproductive-aged women in Maharashtra, suggesting that hypertension may act both as a risk factor and a postoperative complications. These findings underscore the need for comprehensive preoperative screening and careful postoperative monitoring of blood pressure. Addressing socioeconomic and rural–urban disparities through targeted public health interventions is essential to improve gynecological care and reduce long-term cardiovascular risks.

## Introduction

Hysterectomy is the process of removing the uterus, indicated as a definitive treatment for conditions like uterine cancer, fibroids, endometriosis, uterine prolapse, and chronic pelvic pain. Total hysterectomy includes removal of the uterus and cervix with or without removal of ovaries; the cervix is not removed in subtotal hysterectomy (1) while radial hysterectomy involves the removal of the uterus, cervix, part of the vagina, and the surrounding ligaments (2). It is second most common surgical procedures performed on women after caesarian section (3). Excessive menstrual bleeding followed by fibroids have emerged as the leading causes of hysterectomy (4). Positive effects of surgery include relief from excessive bleeding and pelvic pain leading to improvement in the quality of life. The procedure can be performed through abdominal, vaginal, or minimally invasive laparoscopic techniques, while abdominal hysterectomy has been used for endometriosis, adhesions and enlarged uterus while vaginal hysterectomy is a method of choice during uterine prolapse and menstrual abnormalities in normal size uterus (4) whereas laparoscopic hysterectomy is a minimally invasive procedure performed using a laparoscope. Abdominal and vaginal hysterectomies are the most common routes preferred but laparoscopic hysterectomy is one of the minimal access methods that are now being used more often for hysterectomies (5). Hence, the decision regarding type of hysterectomy surgery and its route should be made after considering the underlying condition and patient-specific factors.

In current times, there is an increase in number of hysterectomy surgeries and declining median age for hysterectomy (34.6 years) (6) of patients undergoing this surgery (7), as many women are undergoing this procedure before the age natural menopause. Unnecessary hysterectomies are being conducted among women due to factors like fear instigated by medical professionals, solution for menstrual problems and related taboos, failure of appropriate gynecological care, practical difficulties in living with reproductive health problems, belief that hysterectomy is the best treatment and inappropriate use of insurance (3). While hysterectomy helps treat many gynecological conditions, it is associated with many adverse effects which have emerged as a critical area of concern. Short term complications include excessive bleeding, prolonged recovery time and higher risk of infection while long-term health outcomes are endocrine and metabolic complications; cardiovascular diseases and hypertension. Females with hysterectomy have an increased risk of CVD compared with the women who haven’t undergone hysterectomy. The risk of stroke is significantly higher in the women with hysterectomy. There is no significant difference in incidences of myocardial infarction and coronary artery revascularization (5). Hysterectomy history was associated with four chronic conditions: hypertension, high cholesterol, diabetes mellitus and bone/joint disease (8).

Hypertension is the leading risk factor of cardiovascular diseases and pre-mature death (9), rising due to ageing populations and changes in lifestyle. It has become a leading cause for both mortality and disability in the Indian population (6). Numerous studies have examined the relationship between hysterectomy and hypertension; of which few of the studies have indicated a positive relationship between hysterectomy and hypertension. Hysterectomy is shown to be positively related to CVD especially ischemic heart disease when performed on women under the age of 50 years (10) and studies have also shown that hysterectomy increases the risk of stroke (11) all of which arises due to increase in the blood pressure.

Pathological link between hysterectomy and hypertension is that hysterectomy with oophorectomy results in the immediate termination of the reproductive function, leading to a decline in the production of sex hormones i.e., estrogen and progesterone. These sex hormones play a crucial role in blood pressure regulation. Estradiol (estrogen) has a blood pressure-lowering effect in women, but its levels rapidly dropping the body after hysterectomy which may lead to an increase in blood pressure. Progesterone too has a similar effect on blood pressure, and its reduced levels may lead to increased blood pressure. Sex hormones also affect fat metabolism hence women experiencing sex hormone decline after hysterectomy may gain weight and develop metabolic syndromes which are risk factors for hypertension. Furthermore, reduction in the estrogen and androgen ratio attenuates the vaso-relaxant effects of estrogens on vessel walls and stimulates vaso-constrictive effects. This promotes vascular stiffness in arteries and the narrowing of blood vessels. These mechanisms may be the possible explanations for the relationship between hysterectomy and hypertension (6).

Although hysterectomy is frequently performed to enhance quality of life, it can also be associated with chronic conditions leading to increased risk to life threatening complications. Thus, the decision to undergo hysterectomy requires careful consideration as the surgery is irreversible. Studies have indicated an association between hypertension and hysterectomy. However, it has not been clear whether pre-existing hypertension predisposes women to higher surgical risks or if hysterectomy itself triggers the development of hypertension. This study aims to examine the association between hysterectomy and hypertension among women by different socio-demographic factors and deduce its contribution.

## Data and methods

### Data

This study used data from fifth round of National Family Health Survey (NFHS-5) which is conducted at different points in time and previous four rounds were conducted in 1991–92 (NFHS-1), 1998–99 (NFHS-2), 2005–06 (NFHS-3), and 2015–16 (NFHS-4). NFHS-5 was analyzing for Maharashtra state of women aged 15–49 and conducted during 2019–21. NFHS is a nationally-representative cross-sectional survey that includes representative samples of households from all over India and undertaken by Ministry of Health and Family Welfare, Government of India and coordinated by International Institute of Population Sciences (IIPS), Mumbai as the nodal agency. The NFHS-5 used a stratified two-stage sampling design, which yielded representative samples of households after applying weights to control for the complex survey design. In NFHS-5, probability proportional to size (PPS) sampling was used to select villages from the rural areas and census enumeration blocks (CEB) from urban areas and it provided district-level estimates of demographic and health parameters and data on various socioeconomic and program dimensions that are critical for implementing the desired demographic and health parameter changes. This survey collected information from a Maharashtra state representative sample of 33,755 women aged 15–49 from 31,643 households (TEXT BOX 1). The participants gave their consent for conducting interviews and the use of the data for research purposes.

Text Box 1 **Contributions to the Literature.**

- It gives state-level evidence from Maharashtra on association between hysterectomy and hypertension among reproductive-aged women using NFHS-5 data.
- Hysterectomy prevalence among hypertensive women is more than twice that of the general female population, indicating a potentially association.
- Key socioeconomic and health disparities, with higher prevalence hysterectomy among older, rural, obese, high-parity, and sterilized women.
- By identifying abnormal uterine bleeding as the leading indication, the study underscores the need for improved access to conservative gynecological care and integrated hypertension screening in pre- and post-surgical management.

### Outcome variable

The outcome variable for the study was hysterectomy among women aged 15–49. Hysterectomy is surgery that involves partial or complete removal of the uterus of a woman. The survey asked four direct questions related to hysterectomy to the women: *(i)* whether the women has undergone operation related to the removal of the uterus; *(ii)* how many years ago this operation was performed; *(iii)* place of surgery and, *(iv)* reasons for the surgery.

### Predictor variables

The socio-economic variables like age, residence, religion, caste/tribe, education, wealth index, children ever born, BMI and sterilization status were taken as co-variates.

### Statistical analysis

The analysis of data was used univariate, bi-variate, and logistic regression to fulfill the objective. The sample included women aged 15–49. At the univariate level, the hysterectomy performed by health insurance and reasons for hysterectomy among women were analyses. At the bi-variate level, prevalence of hysterectomy with hypertension women, percentage distribution of place of hysterectomy and logistic regression were performed to examine the statistically significant relationship between predictors and response variables. The association of hysterectomy with hypertension among women along with background characteristics, were analyzed by estimating the adjusted odds ratios (AORs) from the logistic regression analysis (Equation 1). The logistic model takes the following general form;

$$Logit\;P=Ln\;[p/(1-p)]=b_{0}+b_{1}x_{1}+b_{2}x_{2}+\ldots \ldots \ldots \ldots +b_{n}x_{n}+e_{i}$$ (1)

For fulfilling the objective, women who have undergone hysterectomy, the Oaxaca decomposition can be applied to examine disparities in clinical outcomes, such as cardiovascular risk, quality of life, postoperative complications, or long-term hormone therapy use, between subgroups (e.g., by socioeconomic status). This approach helps clinicians and policymakers determine whether observed disparities are primarily driven by clinical characteristics or by systemic factors, thereby guiding targeted interventions to improve long-term outcomes in women post-hysterectomy. Oaxaca decomposition technique is used to explain differences in means between two groups by decomposing the gap into within-group and between-group differences in the effect of the explanatory variable. Suppose a variable, *y*, is outcome variable (hysterectomy) of interest. It has two groups, which shall call the poor and the non-poor. The category has been divided by the wealth index of the first three groups and the last two wealth index groups considered poor and non-poor. It assumes *y* is described by a vector of determinants (socio-demographic predictors), *x*, according to a regression model (Equation 2);

$$y_{i}=\{\begin{array}{c} \beta^{\;poor}x_{i}+\;\varepsilon^{\;poor}\;if\;poor\\ \beta^{non-poor}x_{i}+\;\varepsilon^{non-poor}\;if\;non-poor \end{array}$$ (2)

The vectors of *β* parameters added intercepts. In a single regressor, the non-poor is assumed to have a more advantageous regression line than the poor. The gap between the mean outcomes (hysterectomy), *y*^*non−poor*^ and *y*^*poor*^, is equal to (Equation 3);

$$y^{non-poor}-y^{\;poor}=\beta^{non-poor}x^{non-poor}-\beta^{\;poor}x^{\;poor}$$ (3)

Where $x^{non-poor}$ and $x^{\;poor}$ are vectors of explanatory variables (socio-demographic predictors), evaluated at the means for the non-poor and the poor, respectively.

For example, let's assume two $x^{,}s$, $x_{1}$ and $x_{2}$. It can written as following (Equations 4, 5);

$$y^{non-poor}-y^{\;poor}=(\beta^{non-poor}-\beta^{\;poor})+(\beta_{1}^{non-poor}x_{1}^{non-poor}-\beta_{1}^{\;poor}x_{1}^{\;poor})+(\beta_{2}^{non-poor}x_{2}^{non-poor}-\beta_{2}^{\;poor}x_{2}^{\;poor})$$ (4)

$$=G_{0}+G_{1}+G_{2}$$ (5)

So that the gap in *y* between the poor and the non-poor can be assumed as existence due in part to (i) differences in the intercepts (G_0_), (ii) differences in $x_{1}$ and $\beta_{1}$ (G_1_), and (iii) differences in $x_{2}$ and $\beta_{2}$ (G_2_).

## Results

These data-driven insights serve as a valuable foundation for broader public health implications and future interventions. Table 1 provides a demographic and health profile of women who underwent hysterectomy with hypertension in Maharashtra during 2019–21. Women aged 30–39 and 40–49 constitute the majority of hysterectomy with hypertension cases, with 451 and 685 cases respectively, indicating higher prevalence in middle-aged to older women. Women aged 15–29 are less represented (251 cases), suggesting lower hysterectomy rates in younger women. Slightly more women from rural areas (705 cases) underwent hysterectomy with hypertension than from urban areas (682 cases), but both contribute significantly to the total. The distribution suggests hysterectomy is prevalent in both urban and rural settings. The majority are Hindu women (1,076 cases). Muslims (192 cases) and Buddhists/Neo-Buddhists (90 cases) also represent notable proportions. Scheduled caste (204 cases) and other backward classes (435 cases) form substantial groups. Scheduled tribes (142 cases) and others are also represented. The distribution indicates that hysterectomy with hypertension affects women across caste groups. Women with secondary education or higher (781 cases & 218 cases) constitute nearly half. Women with no education (201 cases) also account for a significant portion, showing implications across educational levels. Women from middle (274 cases) and richer (457 cases) economic groups are well represented. The poorest and poorer groups also contribute notably (71 cases and 178 cases respectively). Women with 2–5 children dominate (1,054) of hysterectomy cases. Women with 0 children (115) and 6 or more children (23) are less represented. The majority fall under Normal BMI (570). Overweight (384) and Obese (253) women are also affected, highlighting interactions between body weight and hysterectomy with hypertension. A high proportion (753) of women who underwent hysterectomy were not sterilized and constitute a substantial share.

**Table 1:** Demographic and health profile of women who underwent hysterectomy with hypertension in Maharashtra during 2019–21.

Background characteristics	Women's interviewed	Hysterectomy with hypertension
Age of Women		
15–29	15,494	251
30–39	9,991	451
40–49	8,269	685
Type of Residence		
Urban	16,080	682
Rural	17,675	705
Religion		
Hindu	27,006	1,076
Muslim	3,938	192
Buddhist/Neo-Buddhist	1,963	90
Others	848	29
Caste/Tribe		
Scheduled caste	5,685	204
Scheduled tribe	3,931	142
Other backward class	9,654	435
Other	14,486	606
Education		
No education	3,987	201
Primary	3,363	188
Secondary	19,563	781
Higher	6,842	218
Wealth Index		
Poorest	2,458	71
Poorer	4,988	178
Middle	7,442	274
Richer	9,303	457
Richest	9,564	406
Children Ever Born		
0 Children	9,590	115
1 Child	5,286	195
2–5 Children	18,592	1,054
≥6 Children	287	23
Body Mass Index	32,282	1,338
Underweight	6,712	130
Normal	17,966	570
Overweight	5,581	384
Obese	2,023	253
Sterilization	14,014	774
No	13,613	753
Yes	401	21
Maharashtra	33,755	1,387

Table 2 presents prevalence of hysterectomy with hypertension among women, focusing on the distribution for the place where hysterectomy performed across different background characteristics and presenting adjusted odds ratios (AORs) in Maharashtra during 2019–2021. Women aged 40–49 have the highest prevalence of hysterectomy with hypertension (13.3%) among the age groups listed. The adjusted odds ratio (AOR) shows that women aged 30–39 have a 35 percent lower likelihood of hysterectomy with hypertension compared with women aged 15–29 [OR: 0.65 (0.17–2.53)], suggesting no statistically significant difference but indicating a lower likelihood of hysterectomy with hypertension in this age group than in the youngest group. In contrast, women aged 40–49 are 2.71 times more likely to have a hysterectomy with hypertension than women aged 15–29 [OR: 2.71 (0.78–9.39)].

**Table 2:** Prevalence of hysterectomy with hypertension among women, focusing on the distribution for the place where hysterectomy performed across different background characteristics and adjusted odds ratios (AORs), Maharashtra 2019–2021. .

Background characteristics	Hysterectomy with hypertension	Place of hysterectomy performed			AOR(CI)
		Public	Private	NGO/Trust	
Age of Women					
15–29^®^	1.8	16.1	57.8	26.2	
30–39	2.7	11.2	88.8	0.0	0.65 (0.17–2.53)
40–49	13.3	15.2	78.7	6.1	2.71 (0.78–9.39)
Type of Residence					
Urban^®^	4.9	9.8	90.2	0.0	
Rural	10.6	17.1	73.9	9.1	2.10** (1.09–4.03)
Religion					
Hindu^®^	8.4	13.8	78.8	7.5	
Muslim	7.2	21.5	78.5	0.0	0.63 (0.27–1.43)
Buddhist/Neo-Buddhist	3.7	13.8	86.2	0.0	0.43 (0.10–1.82)
Others	0.0				Empty
Caste/Tribe					
Scheduled caste^®^	4.8	18.5	76.7	4.8	
Scheduled tribe	7.5	16.2	83.8	0.0	1.37 (0.46–4.08)
Other backward class	7.3	10.0	83.4	6.7	1.18 (0.47–2.94)
Other	9.2	16.6	75.9	7.5	1.83 (0.74–4.54)
Education					
No education^®^	13.8	17.3	79.3	3.4	
Primary	13.3	14.9	81.3	3.8	1.26 (0.63–2.49)
Secondary	6.7	14.3	76.5	9.3	0.90 (0.47–1.70)
Higher	1.3	0.0	100.0	0.0	0.17 (0.02–1.44)
Wealth Index					
Poorest^®^	9.5	35.9	64.1	0.0	
Poorer	10.2	17.2	73.9	9.0	1.45 (0.51–4.16)
Middle	10.7	16.7	80.1	3.2	0.92 (0.32–2.63)
Richer	5.2	14.4	68.0	17.7	0.70 (0.23–2.10)
Richest	7.4	7.0	93.0	0.0	1.23 (0.38–4.00)
Children Ever Born					
0 Child	0.0				
1 Child^®^	5.2	14.3	53.8	32.0	
2–5 Children	8.7	14.8	81.3	3.9	0.96 (0.20–4.69)
≥6 Children	28.2	14.9	85.1	0.0	0.58 (0.04–8.06)
Body mass index					
Underweight^®^	3.0	45.5	54.5	0.0	
Normal	7.5	18.8	76.1	5.1	2.30 (0.66–8.07)
Overweight	8.9	8.3	77.8	13.9	3.28* (0.90–11.90)
Obese	11.6	12.6	87.4	0.0	5.22** (1.35–20.11)
Sterilization					
No^®^	10.3	15.0	80.2	4.8	
Yes	13.7	18.6	81.4	0.0	1.13 (0.30–4.17)
Maharashtra	7.8	14.8	79.0	6.3	

® Reference Category, OR, Odds ratio, ** “0.05” and * “0.10”.

Rural women show a higher prevalence (10.6%) compared to urban women (4.9%) for hysterectomy with hypertension. Rural women are 2.1 times more likely to have a hysterectomy with hypertension than urban women [OR: 2.10** (1.09–4.03)], suggesting statistically significant **(***p* < 0.05**)** difference but indicating a higher likelihood of hysterectomy with hypertension in rural than urban after controlling for other variables. Hindu women constitute the majority of cases, with a prevalence of 8.4 percent. Muslim women have a prevalence of 7.2 percent. The AOR of Muslim women [OR: 0.63 (0.27–1.43)] suggests no statistically significant difference in likelihood compared to Hindu women. Scheduled caste women have a prevalence of 4.8 percent, with other caste groups showing slightly higher prevalence. Women with no education and primary education have similar prevalence rates (around 13%–14%). Women with secondary education show a prevalence of 6.7 percent.

For the wealth index, prevalence was high in middle-income groups (10.7%), where adjusted odds also indicating greater likelihood of hysterectomy with hypertension while the lower wealth quintiles group reported the higher prevalence. Prevalence increased with number of children where women with 6 or more children had the highest rate (28.2%). Obese women had a prevalence of 11.6 percent than counter parts. Obese women are 5.2 times more likely to have a hysterectomy with hypertension than underweight women [OR: 5.22** (1.35–20.11)], suggesting statistically significant **(***p* < 0.05**)** difference but indicating a higher likelihood of hysterectomy with hypertension in obese than underweight after controlling for other variables. Sterilized women had slightly higher prevalence (13.7%) but lower odds [OR:1.13 (0.30–4.17)].

**Figure 1:**
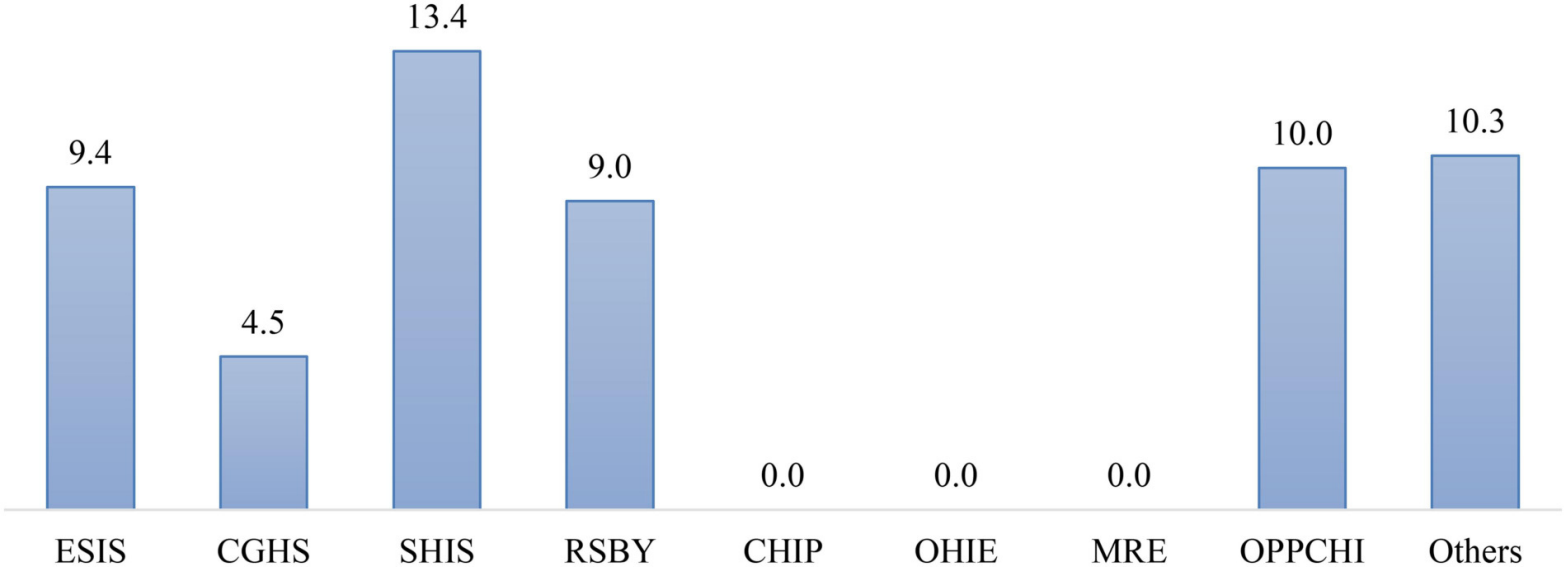
Hysterectomy with hypertension performed by health insurence. ESIS, employees state insurance scheme; CGHS, central government health scheme; SHIS, state health insurance scheme; RSBY, rashtriya swasthya bima yojana; CHIP, community health insurance programme; OHIE, other health insurance through employer; MRE, medical reimbursement from employer; OPPCHI, other privately purchased commercial health insurance.

Figure 1 illustrates the distribution of hysterectomy procedures performed on women with and without hypertension across various types of health insurance in Maharashtra. The figure categorizes the healthcare insurance providers into categories such as ESIS, CGHS, SHIS, RSBY, CHIP, OHIE, MRE, OPPCHI and others. The figure shows that a notable proportion of women undergoing hysterectomy have hypertension, represented by the “Hysterectomy with hypertension” segment, which is distinguished from women who had hysterectomy without hypertension. For instance, women who undergo hysterectomy surgery by different health insurance types utilized amongst which State Health Insurance Scheme (SHIS) was most common and covering about 13.4 percent. Moreover, others insurance schemes included Other Privately Purchased Commercial Health Insurance (10%), the ESIS (9.4%) and RSBY (9%) are also significantly used.

**Figure 2:**
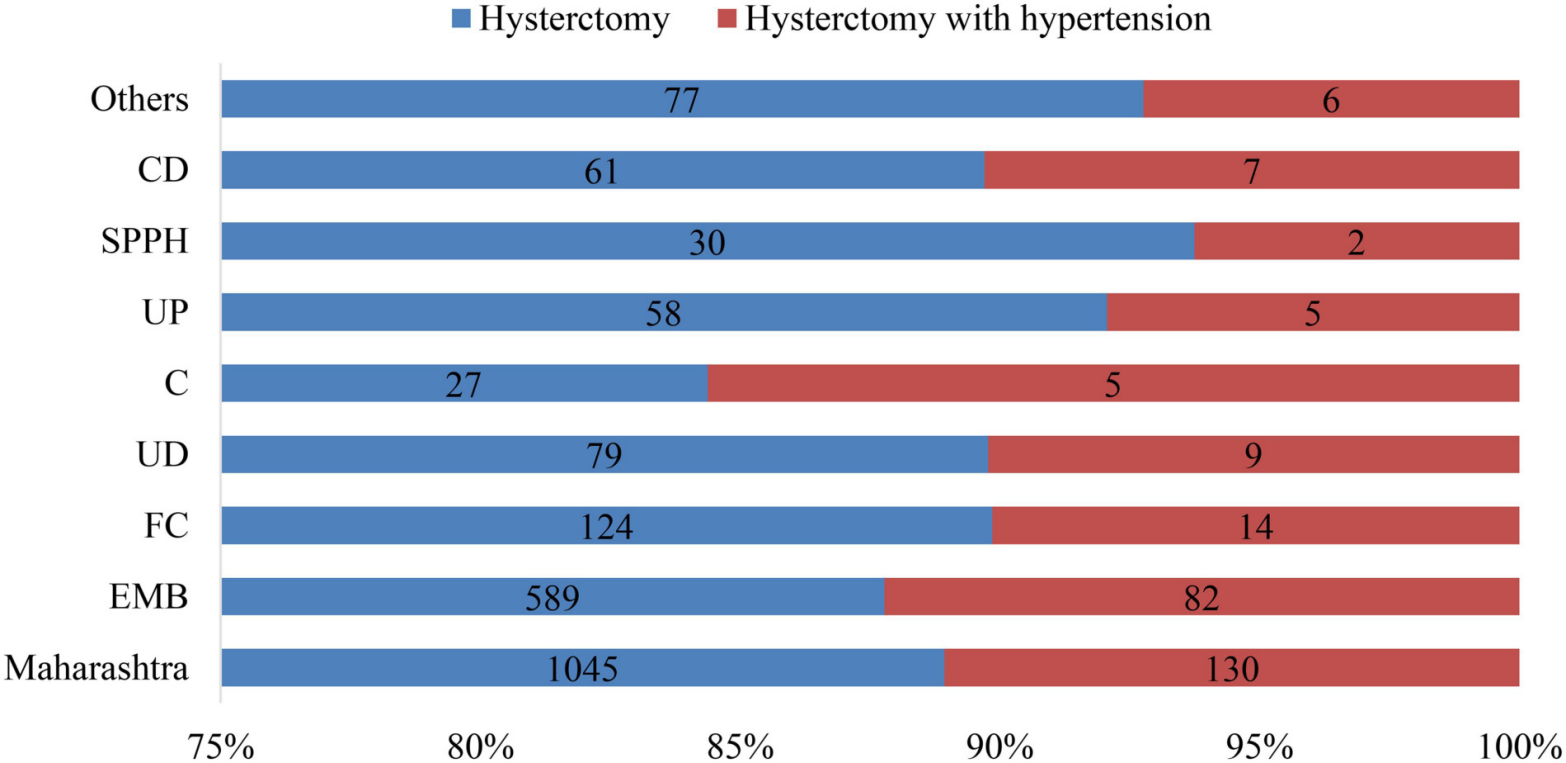
Reasons to performed hysterctomy. EMB, excessive menstrual bleeding; FC, fibroids/cysts; UD, uterine disorder; C, cancer; UP, uterine prolapse; SPPH, severe post-partum haemorrhage; CD, cervical discharge.

Figure 2 illustrates the reasons for performing hysterectomy, comparing cases with and without hypertension among women in Maharashtra. The figure displays the distribution of different reasons categorized under various health insurance schemes or healthcare systems, such as EMB, FC, UD, C, UP, SPPH, CD, and others. The figure presents the proportion of hysterectomy procedures carried out for specific reasons, comparing the two groups: women who underwent hysterectomy with hypertension and those without hypertension. The visual emphasizes that certain reasons for hysterectomy are more prevalent among women with hypertension. For example, the proportion of hysterectomies performed due to conditions related to hypertension [like Excessive Menstrual Bleeding (589) possibly linked to hypertensive complications] might be higher in the “Hysterectomy with hypertension” group (82). Conversely, other reasons such as fibroids, prolapse, or other gynecological conditions are also indicated, with their respective prevalence in each group. The distribution across the various health insurance schemes or health service providers suggests potential differences in the indications for hysterectomy depending on the healthcare system or financing source.

**Figure 3:**
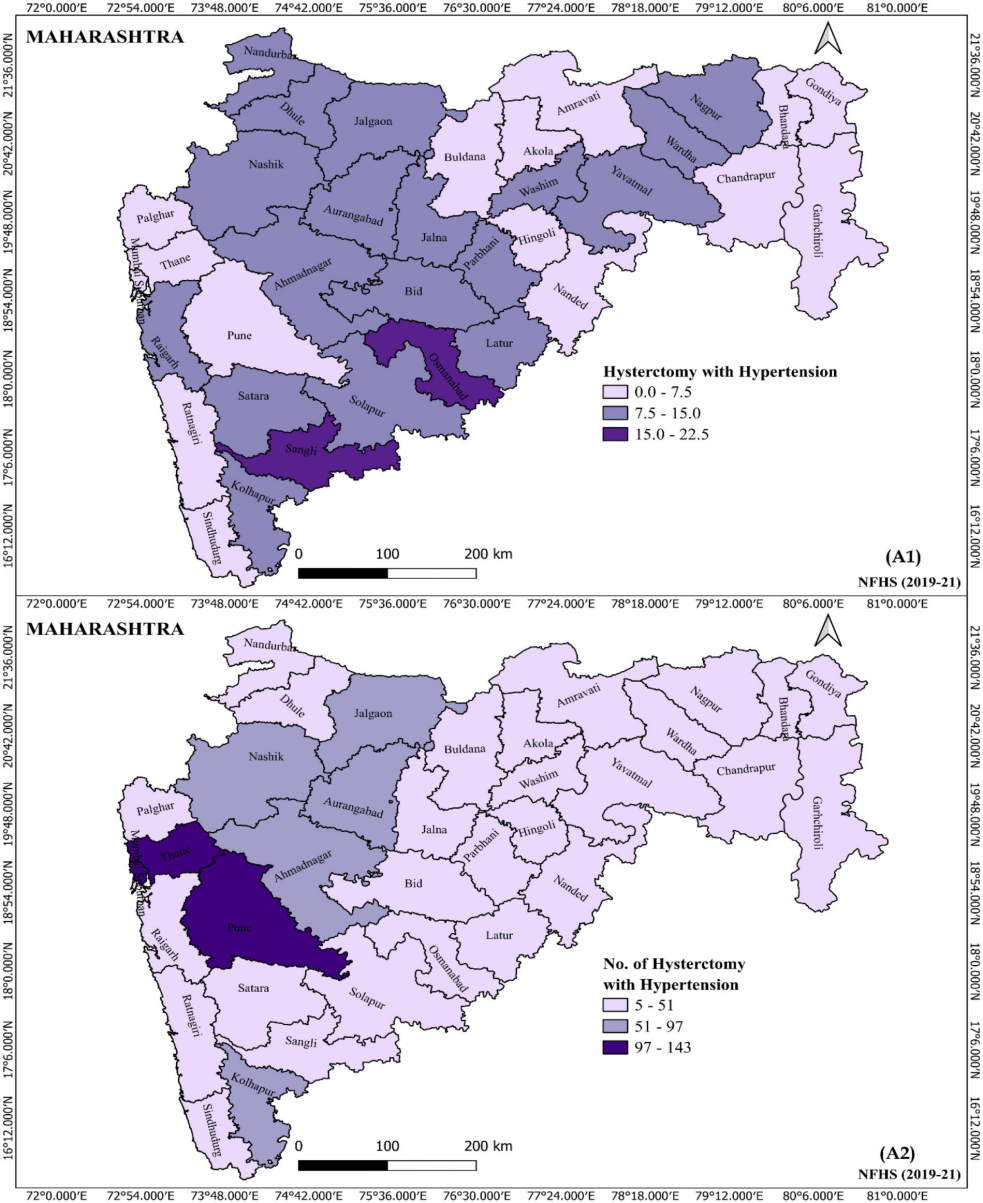
Prevalence of hysterectomy with hypertension among women by different districts, Maharashtra 2019–21.

Figure 3 depicts the prevalence of hysterectomy with hypertension among women in Maharashtra during 2019–21, broken down by different districts. It visually indicates which districts have higher or lower prevalence, highlighting regional disparities. The map or graphical presentation reveals that Osmanabad (22.5%) and Sangli (18.4%) districts have notably higher prevalence rates of hysterectomy with hypertension in dark purple color, suggesting geographical differences possibly linked to local healthcare practices, socio-economic factors, or population health profiles. Conversely, other 16 districts display lower prevalence rates, indicating regional variation in the occurrence or diagnosis of hypertension-related hysterectomy.

**Figure 4:**
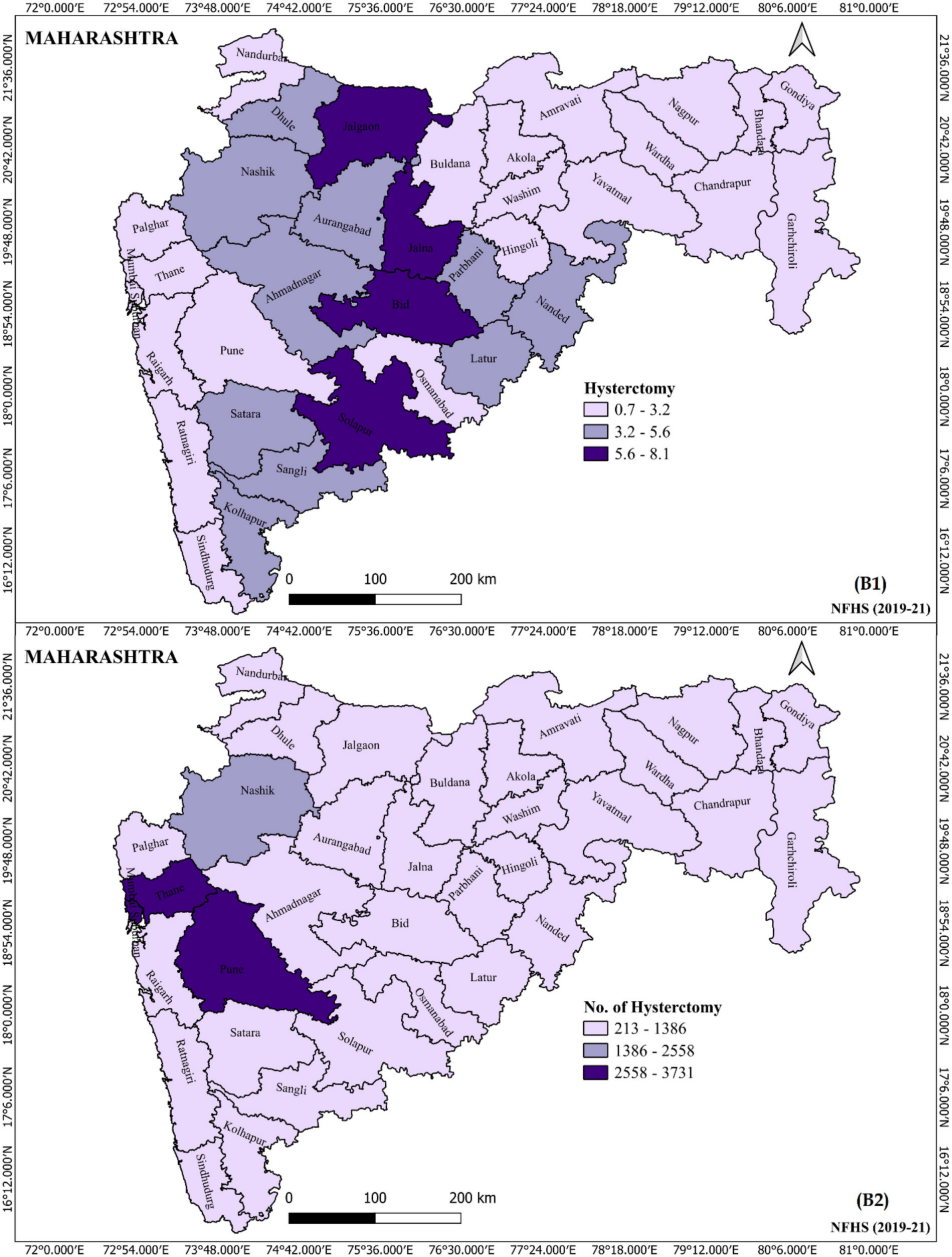
Prevalence of hysterectomy among women by different districts, Maharashtra 2019–21.

Figure 4 illustrates the prevalence of hysterectomy among women across different districts in Maharashtra during 2019–21. The figure provides a geographical visualization of how common hysterectomy procedures are in various districts, highlighting regional differences in prevalence. It identifies four districts [Jalgaon (8.1%), Bid (6.7%), Jalna (6.3%), and Solapur (6.2%)] with higher concentrations of women undergoing hysterectomy, which may suggest variations in healthcare access, socio-economic factors, or cultural practices influencing surgical intervention rates. Other 22 districts show lower prevalence ranges 0.7 percent to 3.2 percent, possibly reflecting differences in healthcare infrastructure, awareness, or population health profiles. The spatial variation underscores the need for district-specific health policies and resource allocation to address underlying causes and improve women's reproductive health services.

Table 3 shows the Blinder–Oaxaca decomposition analysis examining socioeconomic differentials in hysterectomy with hypertension among poor and non-poor women in Maharashtra. Clinically, the prevalence of hysterectomy with coexisting hypertension extracts the inequality between poor and non-poor women. The prevalence of hysterectomy with hypertension was higher among poor women (13.4%) compared to non-poor women (10.3%), resulting in a difference of 3.1 percentage points; however, this difference was statistically insignificant (*p* = 0.283). Although, decomposition technique was performed to see the clinical and socioeconomic factors underlying this difference. The total explained portion 23 percent (−0.0072) was statistically insignificant (*p* = 0.538), suggesting that observable socioeconomic characteristics account for only a small and statistically insignificant share of the poor-non-poor gap. Among the clinical and demographic characteristics, rural women is significantly contributed (coefficient = −0.0192, *p* = 0.017), accounting for 61.8 percent of the explained difference. This suggests that disparities in geographic access to healthcare services, availability of diagnostic facilities, and treatment-seeking behavior between rural and urban populations may influence the coexistence of hysterectomy and hypertension. Overweight and obese women also showed a significant contribution (coefficient = 0.0216, *p* = 0.004), contributing with 69.7 percent to the explained gap, indicating that differences in obesity prevalence between poor and non-poor women may partly explain the observed disparity. From the clinical perspective, obesity is a well-established risk factor for hypertension and may also be associated with gynecological morbidities leading to hysterectomy.

**Table 3:** Contribution of different background characteristics for women who undergone for hysterectomy with hypertension, Maharashtra 2019–21.

Hysterectomy with hypertension	Coeff.	% Contribution	p	95% Confidence Interval	
				Lower	Upper
Differential					
Group 1 (Non-poor)	0.1034		0.0000	0.0779	0.1288
Group 2 (Poor)	0.1344		0.0000	0.0838	0.1850
Difference	−0.0310		0.2830	−0.0877	0.0256
Decomposition					
Endowments	−0.0425		0.2340	−0.1124	0.0275
Coefficients	−0.0265		0.4070	−0.0891	0.0361
Interaction	0.0379		0.3210	−0.0370	0.1128
Difference due to BC (Explained)					
Mothers age (30–49)	0.0016	−5.1	0.3310	−0.0016	0.0047
Rural residence	−0.0192	61.8	0.0170	−0.0350	−0.0034
Hindu religion	−0.0020	6.5	0.5410	−0.0084	0.0044
Others religion	−0.0009	2.9	0.5520	−0.0038	0.0020
ST caste	−0.0024	7.7	0.6470	−0.0126	0.0078
Others caste	0.0059	−19.0	0.2170	−0.0035	0.0152
Mothers with primary	−0.0001	0.4	0.8620	−0.0013	0.0011
Mothers with secondary/higher	−0.0102	32.9	0.1490	−0.0240	0.0037
Normal BMI Mothers	−0.0015	4.8	0.6340	−0.0077	0.0047
Overweight/obese BMI Mothers	0.0216	−69.7	0.0040	0.0071	0.0362
Sterilization	0.0000	0.1	0.9630	−0.0017	0.0017
Total	−0.0072	23.3	0.5380	−0.0302	0.0158
Difference due to coefficients (Unexplained)					
Mother's age (30–49)	0.0587	−189.3	0.4660	−0.0990	0.2164
Rural residence	−0.1004	323.7	0.0200	−0.1849	−0.0159
Hindu religion	0.0220	−71.0	0.8230	−0.1710	0.2150
Others religion	−0.0035	11.2	0.7730	−0.0270	0.0201
ST caste	0.0200	−64.4	0.1580	−0.0078	0.0477
Others caste	0.0220	−71.0	0.6560	−0.0750	0.1191
Mother's with primary	−0.0214	69.0	0.2420	−0.0572	0.0144
Mother's with secondary/higher	0.0019	−6.3	0.9570	−0.0689	0.0728
Normal BMI Mothers	0.0293	−94.5	0.3360	−0.0305	0.0891
Overweight/obese BMI Mothers	0.0199	−64.3	0.4170	−0.0282	0.0681
Sterilization	0.0060	−19.3	0.0750	−0.0006	0.0126
**Constant**	−0.0785	253.0	0.6050	−0.3762	0.2192
**Total**	−0.0238	76.7	0.4250	−0.0823	0.0347

The unexplained component captures (76.7%) differences in the effects (returns) of characteristics between poor and non-poor women. The hysterectomy with hypertension rural women was the only variable showing a statistically significant unexplained contribution (coefficient = −0.1004, *p* = 0.020), accounting for 323.7 percent of the unexplained component. This indicates that the effect of rural residence on hypertension with hypertension differs significantly between poor and non-poor women. Clinically, this may reflect differences in healthcare quality, timing of diagnosis, management of chronic conditions, referral practices, or unmeasured comorbidities across socioeconomic groups. Sterilization status of women showed a marginal contribution 19.3 percent. (*p* = 0.075), which may warrant further clinical investigation. In summary, although the overall socioeconomic gap in hysterectomy with hypertension among poor and non-poor women indicates that the importance of strengthening hypertension screening, obesity management, and equitable access to gynecological and chronic disease care, particularly among socioeconomically disadvantaged and rural populations.

## Discussion

The findings contribute to a growing literature highlighting the health implications of hysterectomy in female populations undergoing this procedure. The overall prevalence of hysterectomy with hypertension among women in Maharashtra is 7.8 percent, with higher rates observed in older “40–49”, rural, less educated, and obese women. It sported by study which shows prevalence of hysterectomy among hypertensive women was nearly twice that of overall general women (TEXT BOX 1), suggesting a possible correlation between the two conditions (6, 7). And a steady rise in hysterectomy rates, with increasingly younger age groups undergoing the procedure frequently before the natural age of menopause (4, 5). According to the National Family Health Survey (NFHS-4), the median age of women undergoing hysterectomy in India is as low as 34.6 years, which is earlier than the age of natural menopause amongst women (3, 4). This early age at surgery raises concerns about long-term hormonal, metabolic, and cardiovascular consequences, particularly in cases involving oophorectomy i.e., removal of ovary (6, 10). The private healthcare sector emerged as a preferred setting for surgery, although Muslim women prefer public health facilities more. Etiologically, endometrial bleeding is the primary cause, however hypertensive women also show higher incidences of hysterectomy for fibroids and uterine disorders. Hysterectomy, a widely performed gynecological surgery, is often indicated for conditions such as uterine fibroids, endometriosis, abnormal uterine bleeding, and uterine prolapse (TEXT BOX 1). It is second only to cesarean section in terms of surgical frequency among women (3).

Several biological mechanisms may underlie this association. Hysterectomy involving oophorectomy leads to a sudden decline in circulating estrogen and progesterone levels, both of which have a vasodilatory effect (10, 11). Estrogen, in particular, modulates vascular tone, enhances nitric oxide-mediated endothelial relaxation, and attenuates vascular smooth muscle proliferation. Its deficiency leads to increased systemic vascular resistance, reduced arterial compliance, and heightened sympathetic activity leading to the development of hypertension (12, 13). Progesterone contributes to blood pressure regulation through aldosterone antagonism and vascular smooth muscle relaxation (13). Therefore, hormonal withdrawal following hysterectomy may disrupt vascular homeostasis, predisposing women to hypertensive disorders over time (10, 11, 13). Beyond hormonal mechanisms, the surgical stress of hysterectomy, post-operative weight gain, and alterations in fat metabolism may further contribute to increased cardiovascular risk (10, 11). Estrogen deficiency has been associated with central adiposity, insulin resistance, and dyslipidemia, all of which are risk factors for hypertension (11, 14). The compounding effect of these mechanisms may suggest the elevated prevalence of hypertension among women undergoing hysterectomy as observed in our study (6, 7).

District-level analysis reveals stark geographic differences, highlighting the need for targeted public health interventions like healthcare education on gynecological health and preventive measures to improve health literacy in women as education and normal BMI emerge as crucial modifiable factors in reducing the burden of unnecessary or early hysterectomies among women. Geographic and socio-demographic analyses revealed a higher prevalence of hysterectomy among hypertensive women in rural districts, with notable differences in prevalence across districts (3, 4). These disparities suggest potential influences of healthcare access and community-level awareness on gynecological health (8). Rural women, often facing limitations in healthcare infrastructure, may resort to hysterectomy as a definitive solution due to lack of access to conservative treatment or diagnostic follow-up (4, 8). These circumstances may influence women to opt for irreversible surgical interventions even in the absence of malignancy or life-threatening conditions (5, 8).

Wealth quintile, parity, and caste also play significant roles in conducting surgery. It is also suggested that hysterectomy is more prevalent among women belonging to lower wealth quintiles and those with lower educational attainment (4, 6, 8). This observation suggests that there is an increase in hysterectomy rates due to less awareness about post-hysterectomy health outcomes (health literacy) and over utilization of the surgical option. Sometimes, limited access to preventive care may drive women toward permanent surgical solutions (4, 5).

It is also found that women amongst hysterectomy with hypertension have State Health Insurance Scheme (SHIS) was most common and covering about 13.4 percent of the sample followed by Other Privately Purchased Commercial Health Insurance (OPPCHI) (10%). The inappropriate use of insurance schemes for hysterectomies has also been flagged in recent years, with reports indicating that financial incentives may influence surgical decisions in both private and public healthcare setups (8, 15). While this study reinforces the hypothesis of a positive association between hysterectomy and hypertension, it also raises questions regarding causality. It remains unclear whether pre-existing hypertension predisposes women to hysterectomy, or if hysterectomy itself contributes to the development of hypertension (6, 7, 16). Some evidence suggests that hypertensive women are more likely to undergo hysterectomy due to complications like uncontrolled abnormal uterine bleeding or endometrial hyperplasia (4, 6). However, if hysterectomy is performed before natural menopause, there is a potential risk for subsequent hypertension and cardiovascular dysfunction (10, 16, 17). This necessitates a more in-depth analysis for appropriate clinical decision-making, where the long-term risks of hysterectomy are carefully weighed against its immediate benefits (12, 17).

Given the irreversible nature of hysterectomy and its potential to impact long-term health, the decision to perform the surgery should be preceded by counseling, exploration of non-surgical options, and a comprehensive risk-benefit analysis tailored to the patient's age, co-morbidities, and reproductive goals (13, 15). There is a pressing need to improve health literacy among women and formulate a set of standardized clinical guidelines to prevent overuse of hysterectomy and improve access to conservative gynecological therapies in resource-limited settings (4, 8, 15).

The cross-sectional nature of this study precludes inference. Furthermore, detailed clinical parameters such as type of hysterectomy (total, subtotal, or radical), time since surgery, etc., were not available (6). Despite these limitations, the study provides important insights into the epidemiological trends and potential cardiovascular consequences of hysterectomy in a large and diverse population cohort (6, 8, 11). Future longitudinal studies are required to explore the pathological mechanisms leading to hypertension after hysterectomy and to assess the cardiovascular outcomes in post-hysterectomy women (10, 11, 17).

## Conclusion

The study reveals that hysterectomy with hypertension predominantly affects women aged 30–49, with similar prevalence across rural and urban areas, and across various caste and socio-economic groups. Women from middle and lower economic backgrounds, those with no or primary education, and obese women show higher prevalence and likelihood of undergoing hysterectomy with hypertension (TEXT BOX 1). The findings also reflect the inequalities in healthcare access, with private facilities being the predominant site for surgeries while public health systems being underutilized. Regional disparities are evident, with districts like Osmanabad and Sangli exhibiting higher prevalence rates. Sociodemographic factors such as rural residence and overweight/obese BMI significantly contribute to inequalities in hysterectomy rates, with socio-economic disparities largely driven by these compositional factors. This emerged as strong contributors influencing the decision to undergo hysterectomy. The preference for surgical intervention over conservative treatment options further highlights gaps in awareness and infrastructure. These insights call for an integrated public health strategy focused on preventive care, informed reproductive choices, and strengthened primary gynecological services to reduce surgical interventions and promote holistic women's health management in Maharashtra.

## Limitations

This study due to its cross-sectional design, which restricts inferences between hysterectomy and hypertension, and reliance on self-reported data (18). The findings are specific to Maharashtra and may not be generalizable elsewhere, while potential confounding factors such as lifestyle and comorbidities were not fully accounted for. Additionally, the dataset does not provide information on the type of hysterectomy (e.g., with or without oophorectomy), surgical approach, or comprehensive clinical indications like antihypertensive treatment, lifestyle factors, and other comorbidities. The absence of longitudinal follow-up limits understanding of long-term health outcomes post-hysterectomy, and the predominance of data from private healthcare settings may introduce selection bias related to socio-economic factors.

## Data Availability

Publicly available datasets were analyzed in this study. This data can be found here: https://www.dhsprogram.com.
